# Spectrocolorimetric evaluation of repaired articular cartilage after a microfracture

**DOI:** 10.1186/1756-0500-1-87

**Published:** 2008-09-23

**Authors:** Koji Hattori, Kota Uematsu, Hiroaki Matsumori, Yoshihiro Dohi, Yoshinori Takakura, Hajime Ohgushi

**Affiliations:** 1 Research Institute for Cell Engineering, National Institute of Advanced Industrial Science and Technology, Amagasaki Site, Amagasaki, Hyogo, Japan; 2 Department of Orthopaedic Surgery, Nara Medical University, Kashihara, Nara, Japan

## Abstract

**Background:**

In clinical practice, surgeons differentiate color changes in repaired cartilage compared with surrounding intact cartilage, but cannot quantify these color changes. Objective assessments are required. A spectrocolorimeter was used to evaluate whether intact and repaired cartilage can be quantified.

**Findings:**

We investigated the use of a spectrocolorimeter and the application of two color models (L* a* b* colorimetric system and spectral reflectance distribution) to describe and quantify articular cartilage. In this study, we measured the colors of intact and repaired cartilage after a microfracture. Histologically, the repaired cartilage was a mixture of fibrocartilage and hyaline cartilage. In the L* a* b* colorimetric system, the L* and a* values recovered to close to the values of intact cartilage, whereas the b* value decreased over time after the operation. Regarding the spectral reflectance distribution at 12 weeks after the operation, the repaired cartilage had a higher spectral reflectance ratio than intact cartilage between wavelengths of 400 to 470 nm.

**Conclusion:**

This study reports the first results regarding the relationship between spectrocolorimetric evaluation and the histological findings of repair cartilage after a microfracture. Our findings demonstrate the ability of spectrocolorimetric measurement to judge the repair cartilage after treatment on the basis of objective data such as the L*, a* and b* values and the SRP as a coincidence index of the spectral reflectance curve.

## Background

Articular cartilage defects do not repair themselves spontaneously [1,2]. Therefore, the poor quality of the repair tissue has led surgeons to develop procedures intended to improve articular cartilage repair. There are several different surgical procedures available to treat cartilage defect, but no method has been judged superior [3]. In clinical practice, surgeons differentiate color changes in repaired cartilage compared with surrounding intact cartilage, but cannot quantify these color changes. If surgeons quantitatively evaluated cartilage color, they can simply understand the determination of surgical effect.

We investigated the use of a commercial spectrocolorimeter and the application of two color models (L* a* b* colorimetric system and spectral reflectance distribution) to describe and quantify articular cartilage. In the present study, we measured the colors of intact and repaired cartilage after a microfracture using a spectrocolorimeter and evaluated the obtained results in comparison with the results of histological, histomorphological and biochemical findings.

## Methods

### Experimental models

A total of 21 adult Japanese white rabbits (3.2–3.7 kg) were used in this study. After anesthesia, the limb was shaved, prepared and draped in a sterile fashion. An anteromedial arthrotomy was performed in the left knee. The patella was dislocated laterally and the patella groove was exposed. The cartilage was resected with a chisel to create a 5-mm diameter defect down to the subchondral bone. The depth of the defect was set to the level at which increased resistance was encountered after the cartilage had been penetrated and the subchondral bone was exposed. Subsequently, a microfracture technique was used in the defect [4,5]. The wound was closed in layers with 2-0 vicryl sutures. No casts were applied to the lower leg. The right knee was left without treatment as a control (group C; n = 19).

The animals were observed during their recovery from anesthesia. Two rabbits were excluded from this series, since one showed signs of infection and the other showed signs of patella dislocation. The remaining animals showed no signs of discomfort. The rabbits were sacrificed at 2 (group M-2; n = 5), 4 (group M-4; n = 7) and 12 (group M-12; n = 7) weeks after the operation with an overdose of phenobarbital sodium salt.

### Spectrocolorimetric measurements

Our spectrocolorimetric evaluation method was described in detail in a previous manuscript [6]. In brief, spectrocolorimetric examination was performed by using a commercial spectrocolorimeter (X-Rite SP64; X-Rite K.K., Tokyo, Japan) driven by a software program (Color/Reader I; Color Techno System Corp., Tokyo, Japan). The measurement area of spectrocolorimeter was 4 mm in diameter. The measurement conditions of spectrocolorimeter were as follows. The reference illumination was D 65 (standard daylight), the geometry was d/8, the incident light was diffuse and the observation angle was 10°. The X-Rite SP64 was positioned with minimal pressure perpendicular to the cartilage defect area or the intact cartilage area as a control. Three consecutive measurements of the L*, a* and b* values and the spectral reflectance ratio per site were averaged for each cartilage measurement. The achromatic luminance signal, L* value represents the relative brightness from black (0) to white (100). The chromatic parameter, a* value represents the color spectrum from green (-) to red (+). A second chromatic parameter, the b* value represents the color spectrum from blue (-) to yellow (+) [7]. Regarding the other index of cartilage color evaluation, the spectral reflectance distribution was automatically calculated at 10-nm wavelength intervals from 400 to 700 nm.

As a coincidence index for the spectral reflectance distribution of the repaired cartilage with respect to intact cartilage, the spectral reflectance percentage (SRP) was determined. The SRP was individually calculated for each animal. The SRP is expressed by

$$\text{SRP}=\int_{400}^{700}f(x)dx/\int_{400}^{700}g(x)dx\times 100(\%)$$

y = f (x), y= g (x), x: wavelength, y: reflectance ratio

where f(x) is the numerical formula of the repaired cartilage sample in the spectral reflectance graph (left knee) and g(x) is the numerical formula of the intact cartilage sample in the spectral reflectance graph (right knee).

After the investigation on repeated measurements of intact cartilage, spectrocolorimetric evaluation was shown to have high reproducibility (the Coefficient of Variation of L*, a*, b* and SPR were 0.0071, 0.024, 0.015 and 0.078 respectively).

### Histological analysis, scoring and biochemical analyses

After the spectrocolorimetric evaluation, each cartilage sample was cut into two pieces along a sagittal plane. One part was used for histological analysis and the other part was subjected to biochemical analyses as bellows.

(1) Histolorical analysis: Sagittal sections (5 μm thick) stained with hematoxylin and eosin, toluidine blue and Safranin-O.

(2) Histological score: the semiquantitative histologic grading scale described by Caplan et al. [8] (Table 1).

**Table 1:** Semiquantitative histologic grading scale

Category	Points
Cell Morphology	
Normal	4
Mostly hyaline cartilage	3
Mixed hyaline and fibrocartilage	2
Mostly fibrocartilage	1
Some fibrocartilage, mostly nonchondrocytic cells	0
Reconstruction of subchondral bone	
Normal	3
Reduced subchondral bone reconstruction	2
Minimal subchondral bone reconstruction	1
No subchondral bone reconstruction	0
Matrix staining	
Normal	4
Slightly reduced	3
Reduced	2
Significantly reduced	1
No staining	0
Filling of defect	
100%	2
50 or 150% (overfill)	1
0%	0
Surface regularity	
Regular, smooth	1
Irregular	0
Bonding	
Both graft edges bonded	2
One graft edge bonded	1
Neither edge bonded	0

(3) Biochemical analyses: Water, proteoglycan (chondroitin sulfate) [9] and collagen (hydroxyproline) contents [10].

### Statistical analysis

Differences among the groups were analyzed using the non-parametric Mann-Whitney U-test. The significance level was set at *P *< 0.05. The relationship between spectrocolorimetric data and the biochemical data were analyzed using the non-parametric Spearman's rank-order correlation method. The significant level was set at *P *< 0.05

## Results

### Macroscopic findings

In group M-2, all of the cartilage defects were filled with reddish tissue (blood clot) with an irregular surface. Some of the defects had white material at the center. In group M-4, most of the defects were incompletely filled with repair tissue that ranged from glistening white tissue (partly reddish) to dull gray tissue. In group M-12, the defects were completely filled with glistening white cartilage-like repair tissue that resembled the normal surrounding articular cartilage. The surfaces of the repair tissue visually differed from smooth to rough.

### Spectrocolorimetric measurements

#### L* a* b* colorimetric system

**Figure 1:**
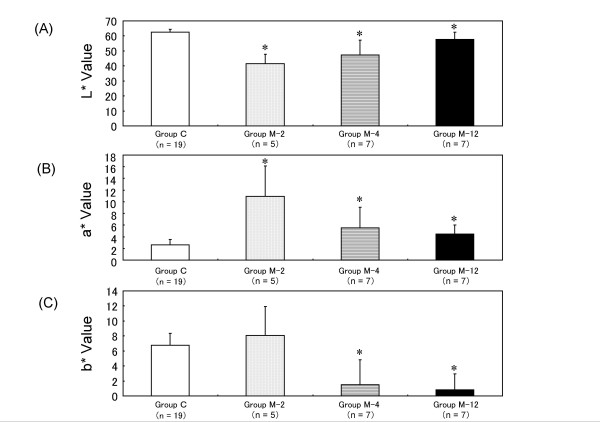
The L*, a* and b* values of the four experimental models. Error bars represent the standard deviation of each group. *P < 0.05 vs. the control group (Group C) by the non-parametric Mann-Whitney U-test.

The differences in the L*, a* and b* values of intact and repaired cartilage are shown in Figure 1. The L* value was decreased at 2 weeks after the operation compared with the control value, and then gradually increased from 4 to 12 weeks after the operation. The a* value was increased at 2 weeks but then decreased to a value close to the control value at 12 weeks. The b* value was not significantly changed at 2 weeks compared with the control value, and then remarkably declined from 4 to 12 weeks.

#### Spectral reflectance distribution

**Figure 2:**
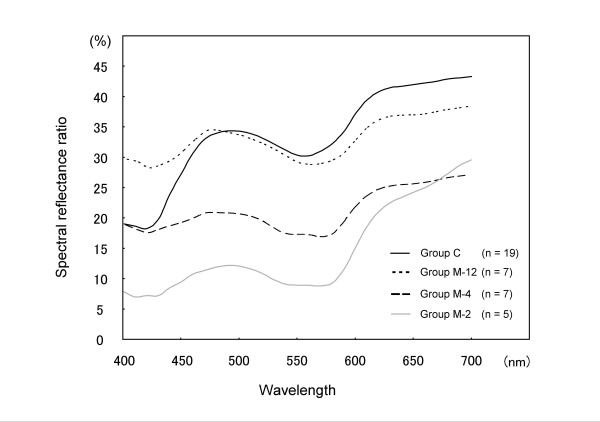
The spectral reflectance curves of the four groups.

**Figure 3:**
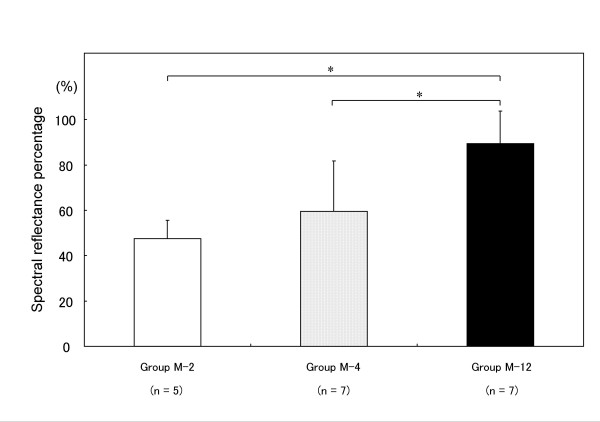
**The spectral reflectance percentages (SRPs) of the three groups**. SRP is as a coincidence index of the spectral reflectance distribution of the repaired cartilage with respect to intact cartilage. *P < 0.05 by the non-parametric Mann-Whitney U-test.

Typical examples of the spectral reflectance curves for groups C, M-2, M-4 and M-12 are shown in Figure 2. The spectral curves of all the groups showed two dips at 420 and 560 nm and a specific peak around 490 nm. There was a gradual increase in the spectral reflectance ratio from 620 to 700 nm. Across all the measured wavelengths, there was a low reflectance ratio in group M-2 compared with group C and a gradual increase in the reflectance ratio over the time course after the operation. As a characteristic difference, group M-12 had a higher spectral reflectance ratio than group C between 400 to 470 nm. The SRP values (mean ± standard deviation) as a coincidence index of the spectral reflectance curves were 47.5 ± 8.0% in group M-2, 59.4 ± 22.3% in group M-4 and 89.5 ± 14.4% in group M-12 (Figure 3). There were significant differences in the SRP values between groups M-2 and M-12 (*P *= 0.004) and between groups M-4 and M-12 (*P *= 0.01).

### Histological findings

**Figure 4:**
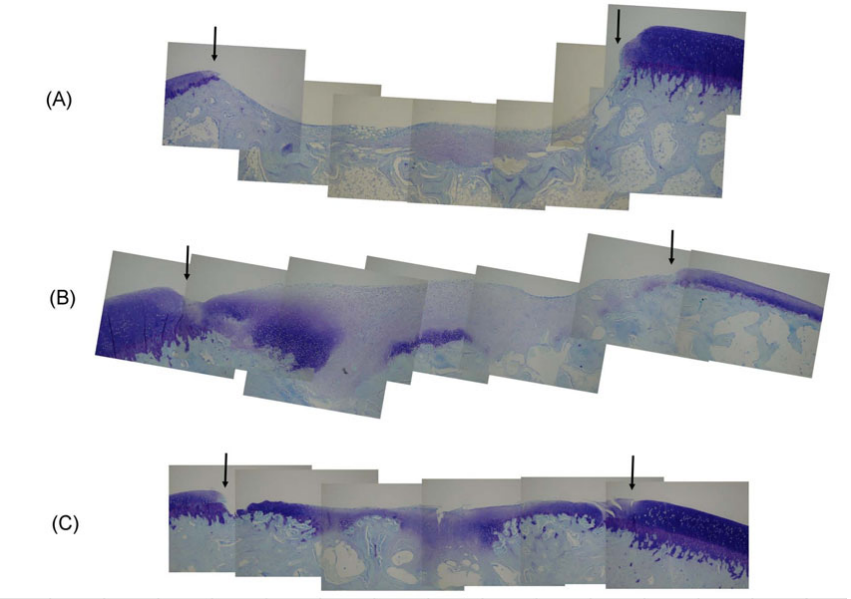
**Photomicrographs of the cartilage defect lesions in rabbits**. Sections at (A) 2 weeks (group M-2), (B) 4 weeks (group M-4) and (C) 12 weeks (group M-12) after a microfracture are shown. Black arrows indicate the borders of the repair site. Toluidine blue staining; original magnification: × 2.5.

Representative toluidine blue-stained sections from the three treatment groups are shown in Figure 4. In group M-2, the defects were not completely filled with newly formed tissue. The lower half of the defects was filled with repair tissue that consisted of fibrous tissue and fibrocartilage. Fibrous tissue was observed in the superficial layer and fibrocartilage was seen in the deeper layer. At the base of the defects, newly formed bone was observed. In group M-4, the defects were filled with three types of tissue, namely fibrous tissue, fibrocartilage and hyaline cartilage. Fibrous tissue was observed in the superficial layer and fibrocartilage was seen in the majority of the defect. Hyaline cartilage occupied the base of the defect or regions near the defect edges. At higher magnification, these cartilaginous areas contained well-differentiated chondrocytes surrounding by metachromatically stained extracellular matrix. In group M-12, the cartilage in the defect was thinner than that in group M-4. The appearance of the cartilage was a mixture of fibrocartilage and hyaline cartilage. The surface of the articular cartilage appeared to be fibrous tissue. The subchondral cartilage seen in group M-4 was completely replaced by new bone.

### Histomorphological findings

The histological scores (mean ± standard deviation) were 1.4 ± 0.5 in group M-2, 4.9 ± 1.8 in group M-4 and 7.3 ± 1.3 in group M-12. Significant differences in the scores were seen between groups M-2 and M-4 (*P *= 0.007), between groups M-2 and M-12 (*P *= 0.004) and between groups M-4 and M-12 (*P *= 0.01).

### Biochemical measurements

**Figure 5:**
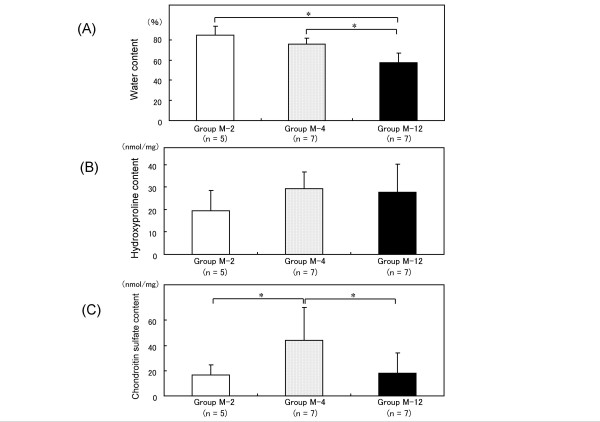
**Biochemical results**. Bar graphs representing the cartilage constitutions in repaired rabbit cartilage. The (A) water contents, (B) hydroxyproline contents and (C) chondroitin sulfate contents of the three groups are shown. *P < 0.05 by the non-parametric Mann-Whitney U-test.

The mean water contents (mean ± standard deviation) were 84.6 ± 8.9% in group M-2, 75.8 ± 6.1% in group M-4 and 57.5 ± 9.5% in group M-12 (Figure 5A). Significant differences in the water contents were found between groups M-2 and M-12 (*P *= 0.007) and between groups M-4 and M-12 (*P *= 0.004). The mean hydroxyproline contents (mean ± standard deviation) were 19.6 ± 8.9 nmol/mg in group M-2, 29.3 ± 7.4 nmol/mg in group M-4 and 27.6 ± 12.6 nmol/mg in group M-12 (Figure 5B). There were no significant differences among the three groups. The mean chondroitin sulfate contents (mean ± standard deviation) were 16.7 ± 8.1 nmol/mg in group M-2, 44.2 ± 25.6 nmol/mg in group M-4 and 18.1 ± 16.0 nmol/mg in group M-12 (Figure 5C). Significant differences in the chondroitin sulfate contents were found between groups M-2 and M-4 (*P *= 0.02) and between groups M-4 and M-12 (*P *= 0.04).

### Relationships between spectrocolorimetoric data and the biochemical data

The L* values was significantly correlated with the water content (*P *= 0.01, r = -0.56). The b value was signigicantly correlated with the hydroxyproline contents (*P *= 0.01, r = -0.58) and the chondroitin sulfate contents (*P *= 0.05, r = -0.46).

## Discussion

Our study attempted to develop constructs for cartilage assessment based on the L* a* b* colorimetric system and the spectral reflectance distribution. During the time course of cartilage repair, the L* value temporarily decreased and then increased to a value close to that of intact cartilage, while the a* value temporarily increased and then decreased to a value close to that of intact cartilage. However, the b* value was decreased at 4 to 12 weeks after the operation. Next, we consider these results from the biological standpoint. Within a defect, a fibrin clot forms after penetration of the subchondral bone, and cells from the blood and bone marrow bound within this clot start to form islands of primitive cartilage. After 6 to 12 weeks, a layer of fibrous cartilage covers the defect surface. Initially, the matrix consists of collagen type I, which later becomes partially replaced by collagen type II, indicating a more hyaline-like tissue [11,12]. Therefore, it may be hypothesized that the a* value represents the color of the fibrin clot, while the L* value represents the newly formed cartilage. From a biochemical viewpoint, collagens, as the major structural macromolecules of the cartilage matrix, would have large effects on the colorimetric evaluation of repaired cartilage. In the present study, the collagen contents were almost constant after the operation. Therefore, the type of collagen, but not the collagen content, is effective for spectrocolorimetric evaluation. Moreover, from the histological findings that most repaired cartilage was a mixture of fibrous tissue and fibrocartilage, it may also be presumed that the b* value reflects the composition of the matrix. However, it is still difficult to reach this conclusion definitely, and the spectrocolorimetric evaluation of articular cartilage requires further investigation.

There is disagreement within a part of the spectral reflectance curves for repaired and intact cartilage. The reflectance ratio is higher in repaired cartilage than in intact cartilage for wavelengths of 400 (purple) to 470 (blue) nm. Moreover, there is a similarity in the spectral reflectance curves (minima at 420 and 560 nm) between groups M-4 and M12. Let us discuss the possible reason for higher reflectance ratio for the repaired cartilage compared with intact cartilage. The optical properties of the cartilage are determined by the surface, the thickness of the individual layer, the content of light-scattering tissue structures and the presence of chromophores and their distribution. Therefore, the differences of cartilage tissue structure showed as the difference of spectral reflectance curve. Thus, the spectral reflectance distribution could help to answer the important clinical question of whether repair tissue is mainly hyaline cartilage or mainly fibrocartilage. However, it is not known the behavior of light in cartilage tissue in detail. We should investigate more relationship between cartilage tissue structure and the behavior of light in cartilage.

In the field of dermatology and plastic surgery, there are several reports on the use of spectrocolorimeter for medical research [13-15]. However, in addition to our earlier study [6], there was no published study about spectrocolorimetric evaluation of articular cartilage. In our series, spectrocolorimeter was applied for assessing the cartilage repaired by two methods, microfracture technique and autologous osteochondral grafting. Using spectrocolorimeter, various surgical methods for cartilage regeneration can be assessed in a quantitative manner. The spectrocolorimeter may be suitable for in situ reliable examination for tissue-engineered cartilage research.

## Abbreviations

SRP: the spectral reflectance percentage.

## Competing interests

The authors declare that they have no competing interests.

## Authors' contributions

KH conceived the study, participated in its design and performed all the experiments. KU performed the histological assessments. HM and YD performed the animal study. YT and HO participated in the design of the animal study and check the manuscript. All Authors read and approved the final manuscript.
